# Editorial: Treatment of animal diseases with veterinary phytotherapy

**DOI:** 10.3389/fvets.2023.1171987

**Published:** 2023-04-06

**Authors:** Fazul Nabi, Dayou Shi, Qingxia Wu, Dost Muhammad Baloch

**Affiliations:** ^1^Faculty of Veterinary and Animal Science, Lasbela University of Agriculture, Water and Marine Sciences, Uthal, Balochistan, Pakistan; ^2^Department of Traditional Chinese Veterinary Medicine, College of Veterinary Medicine, Southwest University, Chongqing, China; ^3^College of Veterinary Medicine, South China Agricultural University, Guanzghou, China; ^4^Animal Science College, Tibet Agriculture and Animal Husbandry University, Linzhi, China

**Keywords:** traditional Chinese medicine, animals disease, animal health, plant, phytotherapy, animal nutrition

The authors have been invited to serve as Guest Editors for this Research Topic. As a Guest Editors we are responsible for identifying the emerging trends, highlights the important topics, or presenting a particular perspective on a subject. Additionally, we ensured the quality and relevance of the publication, as well as bringing fresh ideas and diverse viewpoints to the readers. Phytotherapy, also known as herbal medicine or botanical medicine, is a type of complementary and alternative medicine that uses plant extracts and other natural substances to treat various health conditions (1). These herbs are believed to have various medicinal properties that can help to alleviate symptoms of different health conditions, such as anxiety, depression, insomnia, pain, and inflammation (2, 3). With the advent of antibiotic resistance phytobiotic gain much attention among researchers as an alternative treatment option for livestock, poultry, and aquaculture. Some commonly used herbs in phytotherapy include herbs and their derivatives, plant derived bioactive compounds including lycopene, carotenoids, L-theanine, fucoidan, and humic acid (4, 5). Herbal medicine used in veterinary practice has been gaining popularity as an alternative or complementary treatment option for a wide range of conditions in animals, and it's great to see that there is ongoing research in this field (6, 7).

Numerous natural phytochemical compounds found in plants have medicinal properties that have been used for centuries to promote healing and prevent disease. In veterinary and medical clinical practice, herbal medicine is used in a variety of ways. For example, some veterinarians may use herbal remedies to treat animals with conditions such as bacterial infection, oxidative stress and digestive problems. Similarly, some veterinarian recommended in-feed herbal supplements as an alternative source of antibiotic growth promoter to promote or maintain the productivity of domestic animals. The herbal medicine used for therapeutic tool should be more natural and less harmful than conventional pharmaceutical drugs, it is important to note that not all herbal remedies are safe or effective (8–10). Therefore, the main objective of this Research Topic is to provide the new insight regarding the beneficial application of medicinal plant as source of phytobiotic for the treatment of pathophysiological disorder in veterinary practices. Studies considered for publication in this topic have been classified into following sections.

## Anti-inflammatory potential of phytobiotics

Inflammation is a natural response of the body to injury or infection. However, chronic inflammation can lead to various diseases such as arthritis, diabetes, and cardiovascular disease. Phytobiotics are the natural compounds derived from plants that are known to have various beneficial effects on human and animal health. One of the benefits of phytobiotics is their anti-inflammatory properties. Phytobiotics can help to reduce inflammation by inhibiting the production of inflammatory cytokines and enzymes and by scavenging free radicals that contribute to oxidative stress (11). Examples of phytotherapeutic compounds with anti-inflammatory properties include curcumin, found in turmeric; resveratrol, found in grapes and red wine; quercetin, found in fruits and vegetables; and omega-3 fatty acids, found in fish and some plants.

Several studies have been reported that phytotherapeutic compounds, which are derived from plants, have been found to have anti-inflammatory properties that can help to regulate the body's proinflammatory mediators. These compounds can act on various pathways involved in inflammation, including the inhibition of proinflammatory cytokines, such as tumor necrosis factor-alpha (TNF-alpha), interleukin-1 beta (IL-1beta), and interleukin-6 (IL-6) (12). It has been reported that chronic inflammation has been linked to an increased the risk of cancer development and progression. Inflammation is a complex biological process that involves the activation of immune cells, the release of inflammatory mediators, such as cytokines and chemokines, and the production of reactive oxygen and nitrogen species (ROS/RNS) by immune and non-immune cells, they play a role in the transformation of normal cells to the development of cancer cells. Natural compounds such as resveratrol, curcumin, quercetin, and gingerol have been found to have antioxidant properties, which can help to reduce oxidative stress, prevent inflammation, and modulate these signaling pathways, thereby reducing inflammation (13, 14). In recent study it has been reported that the dietary supplementation of traditional Chinese medicine (TCM) formula significantly increased the levels of antioxidant enzymes, such as superoxide dismutase and glutathione peroxidase, and reduced the levels of reactive oxygen species in the liver of piglets compared to the control group. The TCM formula also decreased the expression of inflammatory cytokines, such as interleukin-1β and tumor necrosis factor-α, in the liver (15). Similarly, another study found that treatment with *Jasonia glutinosa* extract resulted in significant reduction in inflammation and oxidative stress markers in the colon tissue of mice with induced colitis. Additionally, *J. glutinosa* extract improved the integrity of the intestinal barrier by reducing the leakage of gut bacteria and endotoxins into the bloodstream (16).

## Antibacterial potential of phytotherapeutic compounds

The beneficial application of herbal medicine has increased over the past decade, and there has been a growing emphasis on natural plant-based therapeutic agents for the treatment and control of microbial infections. Many plants contain bioactive compounds with antimicrobial properties that can help to inhibit the growth and spread of microorganisms. These compounds include alkaloids, flavonoids, terpenoids, and phenolic acids, among others. Research has shown that many herbal medicines have potent antimicrobial properties and can be used as an alternative or complementary therapy to conventional antibiotics. However, it is important to note that the efficacy and safety of herbal medicines may vary depending on factors such as the plant species, plant part used, method of preparation, and dosage.

Several studies have shown that phytobiotics can inhibit the growth of various bacterial species, including those that are pathogenic to humans and animals. For example, the essential oil of oregano has been shown to be effective against several types of bacteria, including *Escherichia coli, Salmonella enterica*, and *Staphylococcus aureus*. Other commonly studied phytobiotics with antibacterial properties include garlic, cinnamon, thyme, and tea tree oil. These substances have been shown to be effective against a range of bacteria, including those that are antibiotic-resistant.

The antibacterial effects of phytobiotics are believed to be due to their ability to disrupt bacterial cell membranes, interfere with bacterial metabolic processes, and/or inhibit bacterial enzyme activity. Additionally, some phytobiotics may stimulate the immune system to help fight bacterial infections. However, more research is needed to fully understand their mechanisms of action and potential side effects.

## Antiviral potential of phytotherapeutic compounds

Mutations in viruses are a natural occurrence and can lead to the emergence of new strains that may have different characteristics than the original virus. In some cases, mutations can lead to the development of resistance to against antiviral agents (17). The development of resistance to antiviral agents is a concern in the field of virology, as it can make it more difficult to control viral infections. To address this, researchers are continuously working to develop new antiviral agents that are effective against a wide range of viral strains, including those that have developed resistance to existing treatments. There has been growing interest among researchers in exploring the potential of phytotherapeutic compounds as effective treatments for viral infections due to their antiviral properties.

Several studies have suggested that certain phytotherapeutic compounds may have antiviral effects, and could potentially be used as alternative or complementary treatments for viral infections in animals. For example, compounds such as flavonoids, terpenes, and alkaloids found in various plants have been shown to possess antiviral activity against number of viruses including influenza, herpes simplex virus, HIV, and coronavirus (18). On the other hand, some studies have shown that compounds found in plants such as echinacea, elderberry, and garlic have antiviral effects and can help to boost the immunological response. However, it's important to note that the efficacy and safety of these phytotherapeutic compounds in treating viral infections in animals still needs to be thoroughly evaluated through clinical trials and further research. Additionally, it's essential to ensure that the use of such treatments is in compliance with applicable laws and regulations.

## Phytobiotics modulate gut microbiota in animals

These microbiotas, also known as gut microbiota or gut flora, is a complex and diversified collection of microorganisms that reside in the gastrointestinal tract of animals, and poultry birds. The microbiota consists of thousands of different microbial species, including bacteria, archaea, viruses, and fungi, which together form a complex ecological system that plays a crucial role in maintaining the health and wellbeing of the host organism. The study of the gut microbiota is a rapidly growing field of research, as scientists continue to uncover the many ways in which these microorganisms interact with the host and influence various aspects of health and disease.

Phytobiotics, have been shown to have a significant impact on the composition and function of the gut microbiota. These compounds can act as prebiotics, which are substances that promote the growth of beneficial bacteria in the gut, or as antimicrobials, which can help to control the growth of harmful bacteria (19). Some examples of phytobiotics include polyphenols, flavonoids, and terpenes, which are found in a wide range of plant-based foods such as fruits, vegetables, herbs, and spices. These compounds can help to maintain a healthy balance of bacteria in the gut, which is important for overall health and wellbeing of host.

There are numerous bacterial species in the intestine that contribute to the maintenance of the body's homeostatic balance. Some of the most well-known bacterial species that play a crucial role in gut health include *Bifidobacterium, Lactobacillus*, and *Akkermansia muciniphila*. These bacterial species have been found to interact with plant medicines or herbs to improve the health of the bowel and balance of the animal's body. In addition, some herbs such as ginger, peppermint, and fennel have been traditionally used to treat digestive issues and have been found to have antimicrobial properties against harmful bacteria in the gut. These herbs may also help to promote the growth of beneficial bacteria, such as *Bifidobacterium* and *Lactobacillus*, and improve gut health.

## Immunomodulatory role of phytobiotics

The immune system is responsible for protecting the body against infections and diseases. Immunosuppressive diseases are a significant concern in livestock and poultry species because they can negatively impact animal health and productivity, leading to economic losses for producers. Immunosuppressive diseases initiated through several factors, including viral, bacterial, and parasitic infections, as well as environmental and managemental stressors such as poor nutrition or overcrowding. These diseases can weaken the animal's immune system, making it more vulnerable to secondary infections and reducing its ability to fight off pathogens (20).

Previous studies have been suggested that phytobiotics significantly regulated the immune system by activating or suppressing certain immune responses. For example, some phytobiotics can stimulate the production of cytokines, which are signaling molecules that helps to boost immune response (21, 22). Other phytobiotics can inhibit the activity of inflammatory cells, such as macrophages and neutrophils, which can reduce inflammation and tissue damage. Phytobiotics can also have antioxidant properties, which can help to protect the immune system from damages caused by free radicals of reactive oxygen species. Additionally, some phytobiotics can stimulate the growth of beneficial gut bacteria, leading to enhance immunological response. Overall, the immunomodulatory role of phytobiotics is an area of active research, and many plant-derived compounds are being studied for their potential therapeutic effects on immune-related disorders.

## Conclusion

In recent years, there has been an increasing interest in the application of TCM in animal husbandry, particularly as a feed additive for poultry and livestock species. It's great to observed that research reports on the application of TCM as a feed additive have contributed to the improvement of the health and economy of poultry and livestock species. The use of phytotherapeutic compounds or extracts from TCM can indeed have positive effects on the health and performance of animals. It's important to continue research and develop new ways to improve animal health and productivity in a sustainable and ethical manner. The contributions of all the participants involved in this Research Topic are greatly appreciated, as their work will help to advance the field and benefit the industry as a whole.

## Author contributions

All authors listed have made a substantial, direct, and intellectual contribution to the work and approved it for publication.
